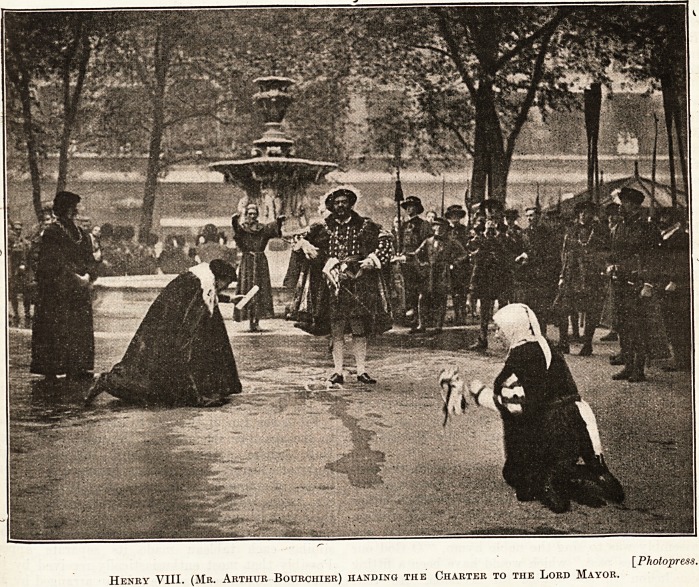# St. Bartholomew's Octo-Centenary

**Published:** 1923-07

**Authors:** 


### ST. BARTHOLOMEW'S OCTO-CENTENARY.

### CELEBRATIONS GRAVE AND GAY.

THE eighth centenary of the foundation of the Priory of St. Bartholomew, in Smithfield, by Rahere for Canons Regular of St. Augustine was celebrated on Tuesday, June 5, and the following days. The ceremonies began with a service in the Priory Church of St. Bartholomew the Great, at which the Bishop of Chester preached. This famous example of Norman architecture is the sole remnant of Rahere's monastic house, and it was fitting that were still threatening. Happily the rain held off, but the uncertainties of the weather had, apparently, decided the authorities to shorten the programme, the whole of which was not carried out. The moment the hospital clock had struck the last note of noon, a stirring fanfare of trumpets broke out from trumpeters of the Coldstream Guards stationed on the roof over the gateway. Then came a sight and a sound which have not been seen or heard at St.

[*Photopress.*

HENRY VIII. (MR. ARTHUR BOURCHIER) HANDING THE CHARTER TO THE LORD MAYOR.

the celebrations should begin within its solemn massive walls, fitting, too, that the service should start with the hymn " Now thank we all our God," and that the lesson should be the majestic " Now let us praise famous men and our fathers that begat us."

At noon the great quadrangle of the hospital was the scene of a " Solemnity " at once truly romantic and unique in the right sense of that much-abused word. The centre of the quadrangle was cleared, and on its fringes congregated a distinguished assembly, many of whom were clad in an academic scarlet which imparted colour to an otherwise grey scene, for the morning had been wet and the skies

Bartholomew's for nearly four centuries, and may very well never be repeated. Into the far end of the quadrangle filed a procession of cross-bearer, thurifer, candle-bearers and priests of the Roman obedience, followed by thirteen Canons Regular of St. Augustine selected from the four Augustinian houses in England, two of whom were Abbots. To Gregorian tones they chanted as they came their hymn in honour of St. Augustine, which begins :—

" Magne Pater Augustine,
Preces nostras suscipe :
Et per eas conditori
Nos placare satage :
Atque rege gregem tuum,
Summum decus Praesulum."

Needless to say, it was sung with the full round vowels, the absence of which makes English public-school Latin unintelligible to all who have not been taught its harsh and grating vocables. The scene was so picturesque that one onlooker likened it to a procession on the stage. Over their spotless white habits the Canons wore cottas; on their heads were birettas, the most hideous of all head-dresses. The Abbots wore black capes and gold pectoral crosses. When the procession reached the centre of the quadrangle it halted near the fountain and, after a prayer, there was a brief Commemoration of St. Augustine, which was heard with difficulty. Then the procession filed back along the other side of the square, chanting the 67th Psalm (in the Roman numeration the 66th), "Deus misereatur nostri." It was all very simple, reverent, and touching, yet highly pictorial and spectacular.

After these solemn and subdued chants, the assembly suffered almost a shock when there marched in, attended by Yeomen of the Guard, a gorgeously-attired herald who, in a marvellous voice, read from an illuminated scroll the command, in the name of the Royal President of the Hospital, the Lord Mayor and Aldermen, the Treasurer and the other Governors, that "the celebrations shall now begin." And begin they did, with a procession of the lame, the halt and the blind, fit subjects for the loving charity of the hospital to be, followed, after a pause, by Rahere on his return from Rome. He meets Richard, Bishop of London, in splendid pontificals, and traces on the ground the outline of the building he wished to raise. The final tableau showed Henry VIII., burly and swaggering (he was represented by Mr. Arthur Bourchier), who, in the midst of Lord Mayor, Aldermen, courtiers and the rowers of the State barge, carrying uplifted oars, granted the Charter of the hospital as it is to-day, and restored the lands which had been alienated when the Priory of St. Bartholomew was suppressed. These finely-produced episodes were admirably "staged" by Mr. Robert Atkins, of the "Old Vic."

So far all was well, but the next item on the programme, "A Procession illustrative of the work of the hospital to-day and of its war services," either did not take place or was not visible to most of those present. Then, according to the programme, the assembly was to sing the noble hymn, "O God our help in ages past," which would have been a fitting conclusion to so notable an occasion. All that happened was that the band of the Welsh Guards played the first verse, nobody sang, and then came the usual few bars of the National Anthem, and all was over. So lame a conclusion was a disappointment to every onlooker. Presumably the proceedings were shortened on account of the weather, yet it remained dry overhead, and the people dispersed unsatisfied and wondering what had happened. A very small choir would have started the singing, and the great volume of joy and thanksgiving would have been heard far outside that noble quadrangle. Those who were present were grateful for the beautiful sights they had seen, and the sweet sounds they had heard, but the anti-climax of the end was a grievous mistake.

The Bartholomew Fair was opened at 1 o'clock on Wednesday by the Lord Mayor. The weather was at its very worst, and a sharp shower descended from leaden skies upon the chilly spectators. There appeared to be considerable uncertainty as to where the ceremony of opening was to take place, and after being hurried from one position to another, many of those present were unable to see or hear anything of what they believed to be going on. However, the unusual experience of being jostled by a Tudor gallant, a hooded friar, or an impressive bearded magician made the wait before business could begin far from tedious. The costumes had been carefully thought out, and in their gaiety and grace they showed their marked superiority over the monotonous masculine attire of to-day. But the spirit of the sixteenth century cannot be recaptured merely by wearing the clothes of the period, and it was well that the medical students have so inexhaustible a supply of energy and fun. It would have been fatally easy for the Fair to have fallen flat, and for its simplicity to have degenerated into dullness. However, all went well, and the booths did an excellent trade.

At "Fairings for Young and Old" piles of ham sandwiches found eager customers in those who were going straight on to the Tableaux at 2.30, and had obviously no time for lunch. There were booths where was sold "Sac and Petum of Virginia," "Toys, Trinketts, Gimcrackes and Staconere," and "Chattels and Phantasies." There was an astrologer who had cast the horoscopes of Anne Boleyn, Cardinal Wolsey and Mr. George "Robie," and there were whipping posts and stocks where many malefactors were made to suffer. On the Thursday Mr. H. A. Saintsbury was to be seen in Richardson's Booth, playing Hamlet, and the Hospital Amateur Dramatic Company were acting "The Play called the Foure P." in the Elizabethan Theatre.

From the fun of the fair it was a change indeed to the spacious solemnity of the magnificent Great Hall, where several tableaux illustrating the hospital's history were given. Arranged by a committee of eminent artists under the chairmanship of Sir Aston Webb, P.R.A., these were magnificent in their stately simplicity. The grouping was perfect, and though the subjects were necessarily somewhat similar each tableau made its separate appeal. Possibly that most enthusiastically received by the very large audience was the tableau arranged by Mr. Solomon J. Solomon, R.A., which showed a cripple girl being healed at the tomb of Rahere. In this tableau particularly those taking part bore themselves with amazing dignity. Other incidents included Rahere, The Courtier, arranged by Mr. Charles Ricketts, R.A.; Rahere in a Dream being delivered from a Dragon by St. Bartholomew, arranged by Mr. Charles Sims, R.A.; Harvey explaining the Circulation of the Blood to Charles I., arranged by Mr. Solomon; and the Hospital's War Work, arranged by Mr. George Harcourt, R.A. The tableaux were explained by Mr. C. S. C. Prance, who is the possessor of a peculiarly clear and beautiful voice, and they were presented with a swiftness which was truly commendable.

## Figures and Tables

**Figure f1:**